# Accuracy of Digital Impression Taking with Intraoral Scanners and Fabrication of CAD/CAM Posts and Cores in a Fully Digital Workflow

**DOI:** 10.3390/ma15124199

**Published:** 2022-06-13

**Authors:** Robert Leven, Alexander Schmidt, Roland Binder, Marian Kampschulte, Jonas Vogler, Bernd Wöstmann, Maximiliane Amelie Schlenz

**Affiliations:** 1 Department of Prosthodontics, Dental Clinic, Justus Liebig University, Schlangenzahl 14, 35392 Giessen, Germany; robert.leven@dentist.med.uni-giessen.de (R.L.); alexander.schmidt@dentist.med.uni-giessen.de (A.S.); jonas.a.vogler@dentist.med.uni-giessen.de (J.V.); bernd.woestmann@dentist.med.uni-giessen.de (B.W.); 2 Dental Laboratory Dental Team, Hauptstrasse 20, 92237 Sulzbach-Rosenberg, Germany; roland.binder@dental-team.de; 3 Department of Diagnostic and Interventional Radiology, University Hospital, Justus Liebig University, Schubertstrasse 81, 35392 Giessen, Germany; marian.kampschulte@radiol.med.uni-giessen.de

**Keywords:** post and core technique, digital dentistry, cad-cam, intraoral scanner, monolithic materials, digital workflow, computer-aided manufacturing, ceramics, polymers, dentistry, prosthodontics

## Abstract

Current intraoral scanners (IOS) enable direct impression taking for computer-aided de-sign/computer-aided manufacturing (CAD/CAM) posts and cores (P+C) with subsequent milling out of monolithic materials. The aim of this in vitro study was to systematically investigate the accuracy of CAD/CAM-P+C in a fully digital workflow, considering different IOS impression methods (Primescan (PRI), Trios4 without (TRI) and with scanpost (TRI+SP)) (Part A), and CAD/CAM milling of zirconium dioxid (ZIR) and resin composite (COM)-P+C (Part B). Five human models were developed in this study. Micro-CT imaging was used as a reference (REF). For Part A, the models were scanned 12 times for each impression method. Then, IOS datasets (n = 180) were superimposed with REF, and scan accuracy was determined using 3D software (GOMInspect). For Part B, one CAD/CAM-P+C (n = 30) was milled for each model, impression method, and material. The triple-scan method was applied using an industrial scanner (ATOS) to determine the accuracy of the fit. Statistical analysis was performed using analysis of variance (ANOVA, *p* < 0.05). Part A showed for PRI significantly lower accuracy than TRI and TRI+SP (*p* < 0.05). The data of Part B revealed significantly higher accuracy for ZIR than for COM (*p* < 0.05). Within the limitations of this study, CAD/CAM-P+C of the ZIR can be recommended for fabrication in a fully digital workflow regarding the accuracy of fit.

## 1. Introduction

Development in computer-aided design/computer-aided manufacturing (CAD/CAM) technology with tooth-colored monolithic materials, and the improvement in intraoral scanner (IOS) accuracy, has led to a wide range of applications in restorative dentistry [1]. Thus, dentists today have the opportunity to manufacture customized posts and cores (P+C) in a fully digital workflow.

Although many treatment situations in modern dentistry no longer require P+C owing to improvements in adhesive technology, there are still clinical situations with a lack of sufficient tooth structure to anchor restorations where P+C is the last resort [2,3].

In contrast to prefabricated posts, customized P+C exhibit a better fit and allow a single restoration combining the P+C [4,5]. However, without CAD/CAM technology, restoration materials for customized P+C were limited to alloy [6]. For aesthetic reasons, P+C alloy are problematic in visible areas. In addition, the topic of corrosion has been discussed in the literature [7]. Furthermore, P+C differ in terms of stress distribution along the root canal [8]. In general, a high stress in the cervical region has been described, which differs depending on the elastic modulus of the P+C [8]. A correlation between failure mode and elastic modulus is discussed, with P+C with a high elastic modulus being more likely to show undesirable failures [7].

Meanwhile, various tooth-colored materials such as zirconium dioxide [9], hybrid ceramics [10], resin composites, polyether ether ketone, and glass-fiber-reinforced composites [5] are available and have already been investigated in a scattered manner for CAD/CAM-P+C [9,11,12]. However, tooth-colored customized P+C are usually composed of zirconium dioxide [13,14]. Their modulus of elasticity is in a range similar to that of alloys [13] and zirconium dioxide offers good biocompatibility [14]. This property can be an advantage during apicoectomies. Zirconium dioxide offers further advantages owing to its corrosion resistance and aesthetic superiority over metallic restorations, and is recommended as a material for P+C [13]. A correlation between fracture resistance and accuracy of fit has been described for zirconium dioxide [15].

Because a good fit of P+C has been described as a decisive factor for a high fracture resistance and retention value [16,17], this aspect is of particular importance.

There are two possible sources of error during the manufacturing of CAD/CAM-P+C: inaccuracies within intraoral scanning of prepared root canals [18], and manufacturing-related inaccuracies due to CAD/CAM milling [17,19].

Nevertheless, regarding impression taking for customized CAD/CAM-P+C, the digital workflow typically begins with the indirect digitalization of a conventional silicone impression or plaster model in a laboratory scanner [11,20], because the IOS previously had a relatively shallow depth of field. To take digital impressions directly in the patient’s mouth using the intraoral scanner, some manufacturers have developed a so-called scanpost (SP), similar to the scanbodies used for implant impressions [5,17]. However, modern IOS shows a higher depth of field, allowing intraoral scans even in areas with undercuts, as described for impression taking in periodontal compromised dentitions [21]. The accuracy of current IOS has been reported to be sufficient for partial-arch impressions in the literature [22,23].

Nevertheless, regardless of the possible scan depth, how accurately the root canal morphology can be transferred to an extraoral model is very important.

Various techniques can be used to measure the accuracy of digital impression acquisition. Thus, superimposition with the reference dataset and a survey of the deviations or a comparison of defined measured values with those of the reference dataset is possible [22,23]. Several methods have been described in the literature for checking the accuracy of the fit of conventional P+C [24,25,26,27]. For example, the replica technique can be used on a patient or indirectly by comparing several impressions. Other methods of measuring the accuracy of fit, such as cutting the tooth and magnifying it under an optical microscope or checking it using micro-computer tomography, are only possible extra-orally, and therefore only suitable after extraction.

To date, there is only one study in the literature that compared the depth of digital impressions and conventional silicon impressions for P+C [18]. There are also few studies on the accuracy of fit of P+C fabricated by taking impressions using IOS with or without SP [5,17,19]. To the best of the authors’ knowledge, no study has investigated the fully digital workflow in one setup by analyzing the possible influence of SP and restoration material.

Therefore, the aim of this in vitro study was to systematically investigate the accuracy of the fit of different CAD/CAM-P+C in a fully digital workflow. The null hypotheses of this study were defined as follows:

(1) There is no significant difference between the different digital impression methods concerning the deviation from the reference dataset (Part A).
(2) There is no significant difference between the different digital impression methods as well as the materials used about the accuracy of fit of CAD/CAM-P+C (Part B).

## 2. Materials and Methods

**Figure 1:**
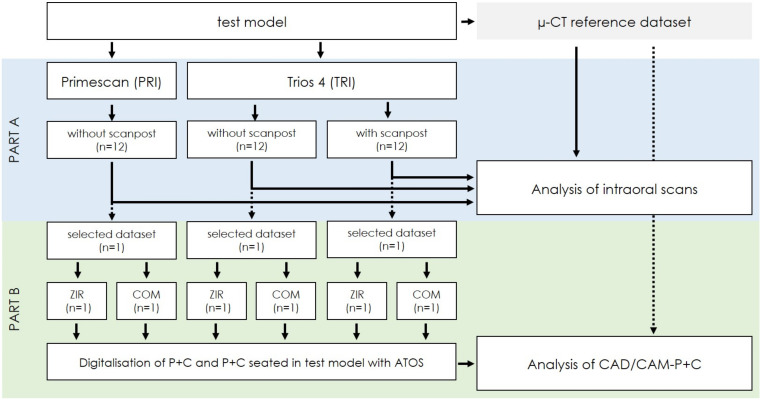
Flow scheme of the investigation; procedure was conducted for each of the five test models.

To systematically investigate possible sources of error in the fully digital workflow for CAD/CAM-P+C, the present in vitro study was divided into two parts: the analysis of the accuracy of digital impression taking with IOS (Part A), followed by an examination of the accuracy of the fabricated CAD/CAM-P+C (Part B). Figure 1 shows a flow scheme of the investigation.

### 2.1. Test Models

To investigate P+C in a common clinical close setup, five test models were constructed using caries-free, sound human teeth that had been extracted for therapeutic reasons in consensus with patients. This study was approved by the local ethics committee of the medical faculty of Justus Liebig University Giessen (Reg. No. 143/09).

For each model, the study tooth, a mandibular premolar with a single root canal, and two adjacent teeth were embedded in a pink-colored acrylic resin. The anatomic crown of the study tooth was removed 2 mm above the cementoenamel junction using a water-cooled high-speed diamond disk. Subsequently, the root canal was prepared by chemo-mechanical root canal preparation using 3.0% sodium hypochlorite for antibacterial irrigation and rotary files (F-360 ISO 15–35, Komet, Lemgo, Germany) for mechanical instrumentation. Finally, the root canal was dried with paper points (Paper Points, Coltène/Whaldent, Langenau, Germany) and filled with gutta-percha (Guttapercha Points, Pluradent, Offenbach, Germany) and sealer (AH Plus Root Canal Sealer, Dentsply DeTrey, Konstanz, Germany) using the cold lateral condensation technique.

**Figure 2:**
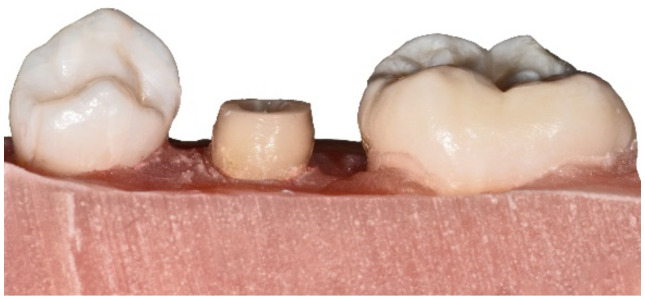
Example of a test model with adjacent teeth and prepared study tooth to receive a P+C restoration.

A post space length of 12 mm and a diameter of 0.9 mm were determined. Post-space preparation was conducted using the ER System (size red, Komet). Additionally, a rotation fuse was burred to prevent rotation of P+C. Finally, a chamfer line was prepared with diamond burrs under constant water cooling, and all edges were smoothed (Figure 2).

### 2.2. Reference Dataset

**Figure 3:**
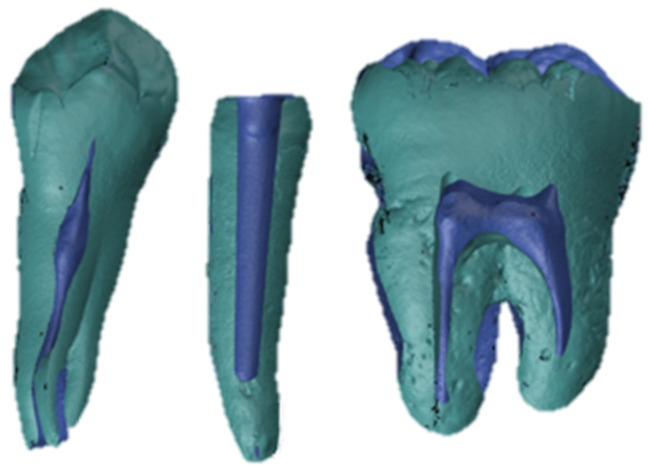
µ-CT reference dataset of a test model with prepared study tooth and adjacent teeth in representative cross section.

A reference dataset for each test model was created by an experienced radiologist (M.K.) using micro-computed tomography (µ-CT, Skyscan 1173, Bruker Optics, Billerica, MA, USA, Figure 3). The tube voltage was set to 130 kV, with a current strength of 60 µA. The models were scanned in a standardized manner, with 1200 slices rotated by 0.2° each. A copper filter with 0.25 mm thickness was added and a structural resolution of 13.5 µm voxel size was reached. Raw data were rendered to a standard tissue language (STL) file using CTAn software (version 1.19, Bruker Optics, Billerica, MA, USA).

### 2.3. Impression Taking with IOS

**Figure 4:**
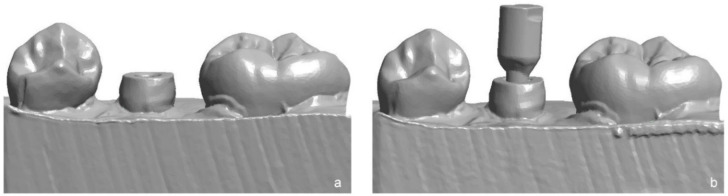
STL dataset of an intraoral scan without (**a**) and with scanpost (SP) inserted in root canal (**b**) for IOS Trios 4.

Two IOS were used for digital impression taking of the test models: Primescan (PRI, version 5.1, Dentsply Sirona, Bensheim, Germany) and Trios 4 (TRI, version 19.2.4, 3Shape, Copenhagen, Denmark). For TRI system, a so-called “post and core” software application was available, first scanning the prepared tooth and then the tooth with a scanpost (SP, Dental Team, Sulzbach-Rosenberg, Germany), inserted in the prepared root canal similar to implant impression with scanbodies (Figure 4).

From each test model, 36 intraoral scan datasets were captured using three impression methods (PRI (n = 12), TRI (n = 12), and TRI+SP (n = 12)), resulting in 180 intraoral scans. Before scanning, the IOS were calibrated with their respective scanning tips according to the manufacturer’s instructions [28,29]. During the scanning process, a standardized ambient light of 500 lx was ensured using a luxmeter (HT309 professional luxmeter, HT Instruments, Korschenbroich, Germany) [30]. The scan path was determined according to the manufacturer’s manual [28,29].

### 2.4. Fabrication of CAD/CAM-P+C

For the fabrication of the CAD/CAM-P+C, one intraoral scan out of the 12 datasets from each of the three impression methods was randomly selected for each test model (n = 5). The design and fabrication of the CAD/CAM-P+C were conducted by an experienced dental technician (R.B.) of an external dental laboratory (Dental Team).

The core height was set to 2 mm and the cone angle was set to 2°. A cube with an edge length of 3 mm was attached to the core. The cement space was set to 80 µm for CAD/CAM-P+C without SP (groups PRI and TRI) and 5 µm for CAD/CAM-P+C with SP (group TRI + SP). The CAD/CAM-P+C were milled on a computer numerical control (CNC) milling machine (CORiTEC 350i, imes-icore GmbH, Eiterfeld, Germany) with a reproduction accuracy of <2 µm. Two restoration materials were investigated (Table 1). After milling, the ZIR was sintered according to the manufacturer’s instructions. A total of 30 CAD/CAM-P+C samples were fabricated.

### 2.5. Analysis of the IOS Datasets (Part A)

**Figure 5:**
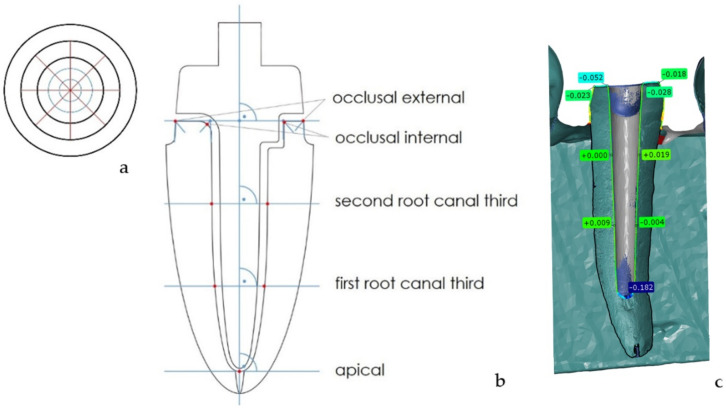
Schematic drawing of measurement points in cross-section (**a**) and longitudinal section (**b**) pooled in five measurement areas (apical, first root canal third, second root canal third, occlusal internal, occlusal external) as well as example of measurement in 3D analysis software (**c**).

To determine the accuracy of the digital impressions, the datasets of the IOS were exported in the STL format. For further analysis, the STL datasets were imported into the 3D analysis software GOM Inspect (version V8 SR1 2020, GOM Inspect, GOM GmbH, Braunschweig, Germany) and superimposed with the respective µ-CT reference dataset using the best-fit algorithm. The deviation of the IOS dataset from the reference was determined at 33 standardized measurement points, and pooled into five measurement areas: apical, first root canal third, second root canal third, occlusal internal, occlusal external (Figure 5).

Therefore, planes were constructed for the reference dataset. Four cross-sections were implemented along the longitudinal axis of the prepared tooth. The first one was in the mesial-distal direction and the other three were at angles of 45°, 90°, and 135° to the mesial-distal direction. Nine measurement points were set for each cross-section. Four points were set as the intersections of the bisectors of the four angles. Two angles were created by the external surface of the tooth and upper surface of the tooth, and the other two by the surface of the prepared root canal and upper surface of the tooth. The line is averaged based on these four points. This line was shifted parallel to one-third and two-thirds of the prepared root canal along the longitudinal axis. Using these parallel shifted lines, another four points on the surface of the prepared root canal were set. The last measurement point was the apical intersection of the prepared root canal surface with the longitudinal axis. This point is the same for all four cross-sect ions.

### 2.6. Analysis of the CAD/CAM-P+C (Part B)

To analyze the accuracy of the fit of the CAD/CAM-P+C, a superimposition according to the triple-scan method was applied [31]. Therefore, all CAD/CAM-P+C were digitized using an industrial 3D scanner (ATOS Core MV 135, GOM, Braunschweig, Germany). Afterwards, each CAD/CAM-P+C was reversibly cemented in their respective test model with a high-viscosity A-Silicone (Fit Test C&B, Voco, Cuxhaven, Germany) at a pressure of 20 N for 2 min [32,33]. The CAD/CAM-P+C seated in the test model was digitized using the same industrial 3D scanner for the digitalization of a single CAD/CAM-P+C.

**Figure 6:**
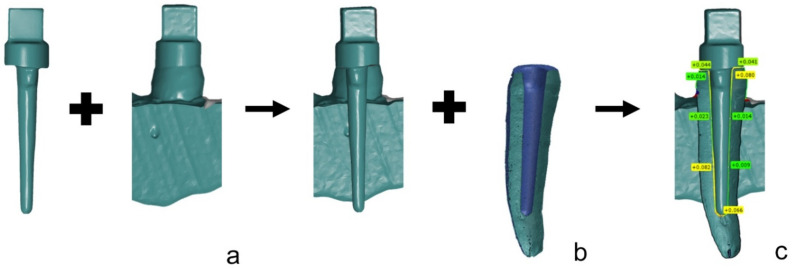
Example of triple-scan-method with superimposition of digitized CAD/CAM-P+C and CAD/CAM-P+C seated in the model (**a**), and subsequent superimposition with µ-CT reference dataset (**b**) to analyze the accuracy of fit at determined measurement points (**c**).

Subsequently, all scans were superimposed for each CAD/CAM-P+C using the 3D analysis software, GOM Inspect. The accuracy of the fit was determined at the same 33 measurement points for the analysis of the scan datasets. Figure 6 shows the separate steps of the superimposition.

### 2.7. Statistical Analysis

SPSS Statistics (version 26, IBM, Armonk, NY, USA) was used for statistical analysis. First, the data of Part A were tested for normal distribution (Kolmogorov–Smirnov). Two-factor analysis of variance (ANOVA) was then performed using the factors impression method (PRI, TRI, and TRI+SP) and measurement area (apical, first root canal third, second root canal third, occlusal internal, and occlusal external). A pairwise comparison of the three impression methods was performed for every level of the measurement area. Due to alpha error accumulation, *p*-values were corrected (Bonferroni).

Second, the data of Part B were tested for normal distribution (Kolmogorov–Smirnov). Subsequently, a multi-factor analysis of variance (ANOVA) was performed using the impression method (PRI, TRI, and TRI+SP), measurement area (apical, first root canal third, second root canal third, occlusal internal, occlusal external), and material (ZIR, COM). Due to the presence of variance heterogeneity, the MIXED procedure was used, and *p*-values were corrected (Bonferroni) due to alpha error accumulation.

The level of significance was set at *p*-value < 0.05.

## 3. Results

Based on the materials and methods section, firstly, the results of Part A regarding the accuracy of digital impression taking with IOS are presented, and then the data of the fabricated CAD/CAM-P+C (Part B) on the accuracy of fit are described. Data from all five test models were pooled, as no statistically significant difference was observed between the models (*p* > 0.05).

### 3.1. Accuracy of Digital Impression Taking with IOS (Part A)

**Figure 7:**
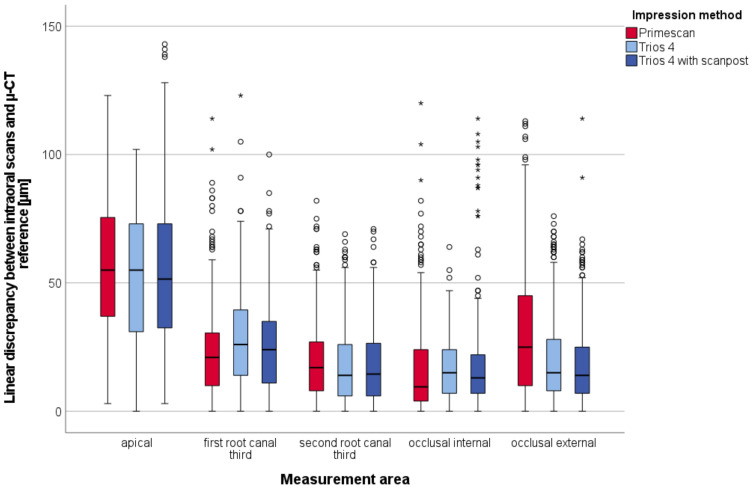
Boxplot diagram of the linear discrepancy (µm) between intraoral scans of all three impression methods and reference µ-CT distributed to the five measurement areas; outliners (O), extreme values (*).

Considering all measurement points, PRI showed significantly lower accuracy than TRI and TRI+SP (*p* < 0.05). Regardless of the impression method, significant differences between the measurement areas were observed (*p* < 0.05). The lowest accuracy was achieved for the apical area. However, upon closer examination, differences between the impression methods and measurement areas could be observed. The linear discrepancy between the three impression methods (PRI, TRI, and TRI+SP) and the respective µ-CT references distributed to the five measurement areas are displayed in Figure 7.

Statistically significant differences between the impression methods were found only in the first root canal third and occlusal external area (*p* < 0.05, Table 2). Except for these two areas, the use of “post and core” software applications could not improve the accuracy of the intraoral scans of TRI. In the occlusal external area, the PRI showed significantly higher discrepancies than the other impression methods.

All impression methods exhibited a high scattering of measurement values.

### 3.2. Accuracy of Fit of CAD/CAM-P+C (Part B)

The two restoration materials for the fabrication of CAD/CAM-P+C revealed a statistically significant difference (*p* < 0.05) with a higher accuracy of fit for zirconium dioxide (Table 3). Regarding the use of scanpost (TRI+SP), significantly higher discrepancies were observed (*p* < 0.05). No significant differences were found between PRI and TRI (*p* = 0.746).

**Figure 8:**
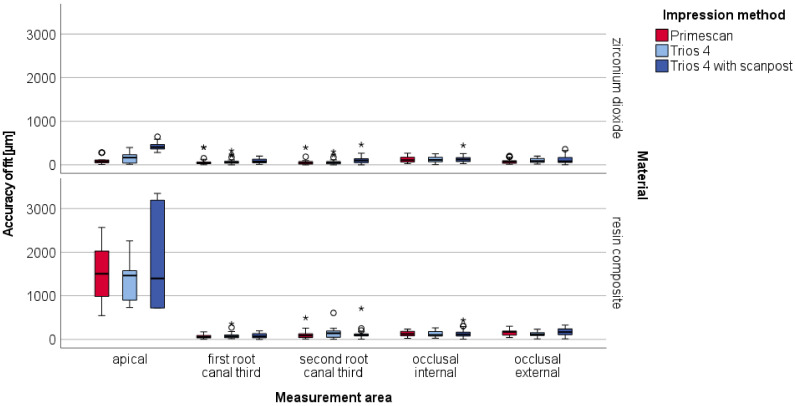
Boxplot diagram of the linear discrepancies (µm) distributed to the five measurement areas for fabricated CAD/CAM-P+C milled of zirconium dioxid and resin composite based on digital impressions with IOS; outliners (O), extreme values (*).

**Figure 9:**
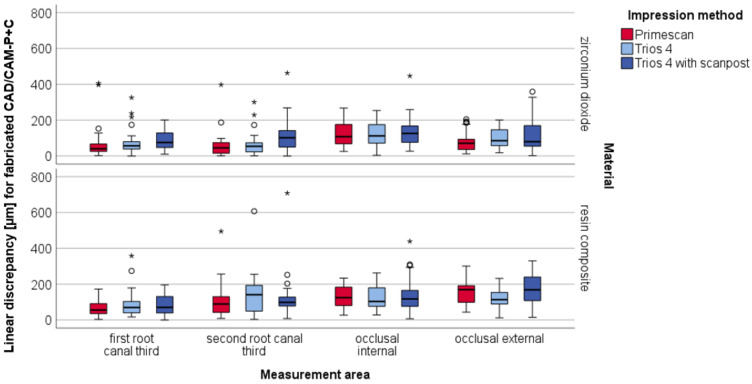
Boxplot diagram of the linear discrepancies (µm) distributed to the four measurement areas (without apical area) for fabricated CAD/CAM-P+C milled of zirconium dioxid and resin composite based on digital impressions with IOS; outliners (O), extreme values (*).

In particular, the apical area of the resin composite exhibited low accuracy, with linear discrepancies of up to 3 mm (Figure 8). For better visibility, Figure 9 shows findings without the apical area.

Statistically significant differences between the impression methods were found in the apical area, first root canal third, and second root canal third (*p* < 0.05, Table 4).

In these areas, the use of an additional scanpost showed a lower accuracy of fit with higher linear discrepancies than the other impression methods.

For the resin composite, statistically significant differences were observed only in the occlusal external area between all impression methods.

## 4. Discussion

The aim of this study was to evaluate the accuracy of the fit of CAD/CAM-P+C based on digital impressions taken by current IOS. The use of IOS for impression taking for CAD/CAM-P+C without a scanpost has been described in a few case reports [34,35]. However, to the best of the authors’ knowledge, no study in the literature has systematically investigated the fully digital workflow considering impression taking with IOS and subsequent fabrication of CAD/CAM-P+C.

### 4.1. Digital Impression Taking with IOS (Part A)

The digital impressions of the IOS showed partly significant differences concerning the measuring areas in relation to the IOS as well as the use of an SP. Considering the overall accuracy of the digital impressions, a significant difference between the Trios 4 and the Primescan could be shown. No significant difference was found for the use of an SP within the Trios 4 groups. Consequently, the first null hypothesis that there is no significant difference between the different digital impression methods with regard to the IOS in impressions for CAD/CAM-P+C in vitro has to be partially rejected.

An in vitro study by Pinto et al. [18] compared digital and conventional impression techniques for CAD/CAM-P+C with regard to impression depth. The investigated area was compared to the apical measurement area in the present study. Pinto et al. described a mean deviation of 19.58% for all digital impressions using the IOS Trios compared to the conventional impression technique [18]. In contrast to these findings, the deviations in the present study were tenfold smaller. This difference might be explained by different generations of IOS; Pinto et al. [18] used the first generation of the IOS Trios, whereas in this study, the latest IOS Trios 4 was investigated. Technical improvements in hardware and software regarding the accuracy of IOS are described in detail in the literature [36].

Gurpinar et al. [37] investigated the influence of endocrown preparation depth on the accuracy of digital impressions and reported a higher deviation for more deeply prepared endocrowns. This coincided with an increase in deviation along the prepared root canal from the occlusal internal to apical measurement areas in the present study. The preparation depth of endocrowns ranged from 2 to 5 mm [37]. Gurpinar et al. reported at 5 mm is within the range determined in this study for the measurement areas of the first and second root canal thirds. Larger scattering in the data can be observed by the deviation of this measurement area in this in vitro study for various reasons. In the in vitro study by Gurpinar et al. [37] the test specimens were manufactured with resin composite, and no human teeth were used. Furthermore, the selection of the IOS differed; even though IOS Primescan was used as well, IOS Trios 3 is the previous generation of the IOS Trios 4 analyzed in this study.

For the occlusal internal and external measurement areas, a comparison of the deviations of a single tooth preparation can be considered, as these areas are at a comparable vertical height. In an in vitro study, Park et al. [38] investigated the accuracy of the digital impressions of six different IOS for complex single-tooth preparations. From their results, they assumed that the deviation of the digital impression increased with the complexity of the preparations [38]. The preparation of a tooth for the post and core is also one of the more complex preparations, which explains why a similar range of mean values for the deviation seems consistent in the comparison.

Haddadi et al. investigated the accuracy of digital impressions of multiple IOSs in comparison to conventional impressions based on single tooth preparation [39]. The measurement area “Prep Margin” can be considered comparable to the occlusal external measurement area. The values for the mean deviation in the study differed depending on the IOS [39]. The two IOSs they used were the IOS Trios 3 and the IOS Omnicam [39], which represent the previous generations of the IOS Trios 4 and IOS Primescan. They determined the mean deviation for these IOSs, which was smaller than that of the respective successor [39]. The use of the IOS and that the accuracy of the current generation of the IOS may have played a role in these differences. However, comparison of the mean values of the studies is difficult because Haddadi et al. used a tooth made of resin composite.

The difference in the deviation from the reference dataset to the two IOS Trios 4 and IOS Primescan can be attributed to several factors. One may be due to the different imaging techniques used for IOS. IOS Trios 4 uses confocal microscopy as its measurement principle [40]. The measurement technique of IOS Primescan is called optical high-frequency contrast analysis [21], a combination of confocal microscopy and fringe light projection, which may have an influence on the accuracy compared to sole confocal microscopy. However, to the best of our knowledge, no study has investigated this factor. Apart from the different imaging techniques, further distinguishing features are the software and individual algorithms. The computing algorithms are not disclosed by the manufacturer. Therefore, it is not possible to judge whether these algorithms have an influence on the results and whether this leads to differences.

There are no studies available on the accuracy of the IOS Trios 4 for single-tooth preparation. For the IOS Primescan, Zimmermann et al. [41] investigated the accuracy of different single-tooth preparations compared to other IOS in an in vitro study. The digital impressions of the IOS Primescan showed a mean deviation for the preparation margin, which corresponds to the occlusal external measurement area, which was approximately 10 μm greater for the mean deviation as well as for the spread width. This also can be explained by different software versions of IOS Primescan. The influences of different versions of the CEREC software on the deviation have been described in the literature [42].

### 4.2. Accuracy CAD/CAM-P+C (Part B)

The accuracy of fit of the CAD/CAM-P+C showed a significant difference regarding the materials. The use of digital impressions from different IOS showed significant differences for the accuracy of fit when considering the measurement areas. The use of an SP showed no significant difference for the accuracy of fit, but when the material zirconia was considered, there was a significant difference. Therefore, this null hypothesis has also been rejected with regard to the material. It has to be partially rejected for the use of an SP as well as for the different digital impressions of the IOS.

Regardless of the impression method, the results of the accuracy of fit of CAD/CAM-P+C showed significant differences between the two monolithic materials investigated.

ZIR was milled in a pre-sintered form with a volume shrinkage of 20% owing to sintering [43]. This circumstance may be a possible factor influencing the accuracy of the fit. However, the CAD/CAM-P+C fabricated using ZIR showed an even higher accuracy of fit compared to COM, which was milled in the final form. When milling CAD/CAM-P+C, small vibrations caused by the milling drills can result in strong forces on the milling material owing to the long lever along the post portion. The more rigid the milling material, the faster the occurrence of fractures or inaccuracies. Furthermore, the fracture resistance of the material decreases with increasing diameter. This was higher when milling the ZIR owing to the subsequent sintering. These two differences in fabrication may be the reason for the poorer fit of the COM CAD/CAM-P+C, suggested by the significantly poorer fit of the apical measurement area. Further studies are required to obtain more precise data regarding the influence of the material on the accuracy of the fit.

The CAD/CAM-P+C created using a scanpost showed significantly poorer accuracy of fit for the ZIR and the lowest values for the COM. One reason for the discrepancy between the results could be that the exact position and path of the root canal could not be precisely determined. Compared to the scan bodies of implants, for which a high transfer accuracy is described in the literature [44], the scanposts are seated solely because of friction with the wall of the prepared root canal and are not firmly screwed in place. Accordingly, the scanpost can always rotate along its axis.

There are no studies in the literature that investigate the accuracy of the fit of P+C based solely on digital impressions of an IOS without a scanpost. Tsintsadze et al. [45] investigated the accuracy of the fit of CAD/CAM-P+C and created a digital impression using a laboratory scanner and determined the accuracy of the fit on the horizontal sections. Compared to the results of the measurement areas, the first and second root canal thirds suggested a better accuracy of fit for the CAD/CAM-P+C in the present study. However, Tsintsadze et al. used an experimental CAD/CAM material [45], which may have influenced the accuracy of fit.

In an in vitro study, Kanduti et al. [46] investigated the influence of the manufacturing method of P+C made of a cobalt-chromium alloy on the accuracy of fit. They fabricated P+C conventionally molded and cast, as well as digitally by an IOS using scanpost and laser sintering of the CAD/CAM-P+C, and found a better fit for the conventionally fabricated P+C [46]. They determined the accuracy of the fit on four horizontal sections reconstructed by superimposing µ-CT data [46]. In comparison, the mean deviations of the accuracy of fit of the CAD/CAM-P+C in the measurement areas of the first and second root canal third were in a similar range. However, the different fabrication techniques of CAD/CAM-P+C as well as the restoration material must be considered. Furthermore, Kanduti et al. stated that they selected the scanpost according to friction in the root canal [46], which did not have to correspond to the preparation drill, so inaccuracies may have arisen. In this study, a scanpost corresponding to the preparation drill was used, which meant that this inaccuracy did not occur.

Additionally, using a µ-CT, Moustapha et al. investigated the accuracy of fit of CAD/CAM-P+C made of a fiber reinforced composite using a scanpost in addition to an IOS [5]. They determined the accuracy of fit marginally, at the internal gap, apically, and horizontal sections [5]. When comparing the accuracy of the fit with that of the present study for individual measurement areas; apically, the CAD/CAM-P+C had an accuracy of fit that was slightly worse than that of the ZIR CAD/CAM-P+C without a scanpost and better than those made with a scanpost [5]. The accuracy of fit of the COM CAD/CAM-P+C has a much worse fit than that of the in vitro study by Moustapha et al. [5]. In the horizontal ranges, the CAD/CAM-P+C in the present study showed better or equal values for the accuracy of fit for ZIR and equal or slightly worse for COM. Regarding the internal gap, equal to the occlusal internal measurement area, the accuracy of fit of the CAD/CAM-P+C in the present study deviated by 10 μm [5]. The same is shown in the marginal measurement area, equal to the occlusal external for the CAD/CAM-P+C made of ZIR. The CAD/CAM-P+C made of COM showed an accuracy of fit that was approximately 50 μm worse than the values determined by Moustapha et al. [5]. With the exception of the apical measurement area, the values for the accuracy of fit were found to be in a similar range.

The accuracy of fit of CAD/CAM-P+C in the apical region was also investigated by Hendi et al. [17]. They compared conventionally cast P+C with CAD/CAM-P+C, which were created using a digital impression and scanpost [17]. Conventional cast P+C showed better fit accuracy [17]. In comparison to the CAD/CAM-P+C, the ZIR CAD/CAM-P+C in the present study showed a slightly better fit, whereas those made of COM showed a much poorer fit. The present report tested CAD/CAM milling of zirconium dioxid and resin composite. Future studies are required to investigate more CAD/CAD materials that can be used in combination with IOS and 3D printing, such as fiber reinforced composites [47] and metals [48].

All ZIR CAD/CAM-P+C in the present study showed an apical distance of less than 2 mm between the CAD/CAM-P+C and gutta-percha. In the literature, this is the cutoff value for assessing whether P+C is applicable [17,19]. Consequently, all ZIR CAD/CAM-P+C in this in vitro study would have been clinically acceptable.

However, some limitations have to be discussed as in vitro studies do not have possible influences from saliva, gingival fluid, blood, and anatomical restrictions. Therefore, in vivo studies are required to confirm the reported findings. Furthermore, it has to be considered that the material selection of the P+C should not be based solely on the accuracy of fit. Other factors such as the elastic modulus have also be taken into account, as P+C with a high elastic modulus are more likely to lead to undesirable failure [7]. P+C made of a material with a similar modulus of elasticity to dentin show more desirable failure [7]. For example, P+C from fiber-reinforced resin composite can be removed by ultrasound [49], which is more difficult for P+C made of zirconia [13].

Another factor for CAD/CAM-P+C is the luting, whether it is adhesive or with conventional cementation. Whereas zirconia P+C can luted adhesively or with cement [14], resin composite P+C always requires adhesive luting [50]. In the case of adhesive placement, differences have been described regarding the retention value depending on the luting material [50]. Whether these differences also exist for CAD/CAM-P+C should be investigated in further studies.

## 5. Conclusions

Overall, within the limitations of this in vitro study, the CAD/CAM-P+C of ZIR can be recommended for fabrication in a fully digital workflow regarding the accuracy of fit. The deviations of all investigated digital impression methods from the reference dataset were within a clinically acceptable range. The use of scanposts is not required for current IOS and results in a lower accuracy of fit of CAD/CAM-P+C compared to intraoral scanning without scanposts.

## Figures and Tables

**Table 1:** Materials for CAD/CAM-P+C.

Abbreviation	Material	Product Name	Brand	Lot Number	Expiration Date
ZIR	3Y-TZP zirconium dioxid	dima Zirconia ST	Kulzer GmbH, Hanau, Germany	26026	2025–07
COM	resin composite	Brilliant Crios	Coltène/Whaledent AG, Altstätten, Switzerland	J72547	2022–12

**Table 2:** *p*-values of pairwise comparison (*p*-value < 0.05, NS = not significant) for the linear discrepancies between intraoral scans of the three impression methods (PRI = Primescan, TRI = Trios 4, and TRI+SP = Trios 4 and scanpost) and µ-CT reference distributed to the five measurement areas.

Measurement Area		TRI	TRI + SP
apical	PRI	NS	NS
	TRI	/	NS
first root canal third	PRI	NS	*p* < 0.001
	TRI	/	*p* = 0.021
second root canal third	PRI	NS	NS
	TRI	/	NS
occlusal internal	PRI	NS	NS
	TRI	/	NS
occlusal external	PRI	*p* < 0.001	*p* < 0.001
	TRI	/	*p* = 0.024

**Table 3:** Descriptive statistics of linear discrepancies (µm) for fabricated CAD/CAM-P+C milled of zirconium dioxid and resin composite based on digital impressions with IOS (PRI = Primescan, TRI = Trios 4, and TRI + SP = Trios 4 and scanpost).

Material	Impression Method	Mean ± Standard Deviation (µm)
zirconium dioxid	PRI	84.57 ± 76.90
	TRI	95.96 ± 77.08
	TRI + SP	144.89 ± 125.90
resin composite	PRI	267.54 ± 502.35
	TRI	259.59 ± 446.63
	TRI + SP	320.51 ± 674.87

**Table 4:** *p*-values of pairwise comparison (*p*-value < 0.05, NS = not significant) of the linear discrepancies (µm) distributed to the five measurement areas for fabricated CAD/CAM-P+C milled of zirconium dioxid and resin composite based on digital impressions with IOS (PRI = Primescan, TRI = Trios 4, and TRI+SP = Trios 4 and scanpost).

Material	Measurement Area		TRI	TRI + SP
zirconium dioxid	apical	PRI	NS	*p* < 0.001
		TRI	/	*p* < 0.001
	first root canal third	PRI	NS	*p* = 0.033
		TRI	/	NS
	second root canal third	PRI	NS	*p* < 0.001
		TRI	/	*p* = 0.005
	occlusal internal	PRI	NS	NS
		TRI	/	NS
	occlusal external	PRI	NS	NS
		TRI	/	NS
resin composite	apical	PRI	NS	NS
		TRI	/	NS
	first root canal third	PRI	NS	NS
		TRI	/	NS
	second root canal third	PRI	NS	NS
		TRI	/	NS
	occlusal internal	PRI	NS	NS
		TRI	/	NS
	occlusal external	PRI	*p* = 0.036	NS
		TRI	/	*p* = 0.023

## Data Availability

The datasets of this article are available from the corresponding author on a reasonable request.
